# Effect of Sodium−Glucose Co‐Transporter‐2 Inhibitor on Estimated Plasma Volume in a Patient With Heart Failure With Reduced Ejection Fraction and a Patient With Heart Failure With Preserved Ejection Fraction

**DOI:** 10.1002/clc.24303

**Published:** 2024-07-19

**Authors:** Andreasová Taťána, Málek Filip

**Affiliations:** ^1^ Third Faculty of Medicine Charles University Prague Czech Republic; ^2^ Center of Nuclear Medicine Faculty Hospital Bulovka Prague Czech Republic; ^3^ Na Homolce Hospital Prague Czech Republic

**Keywords:** biomarker, heart failure, hemoconcentration, plasma volume, sodium−glucose co‐transporter‐2 inhibitor

## Abstract

**Background:**

The increased diuresis after sodium−glucose cotransporter 2 inhibitor (SGLT2i) was associated with a reduction of the estimated plasma volume (ePV) in type 2 diabetic patients.

**Hypothesis:**

We hypothesized that the early effect of SGLT2i on ePV may be monitored by the change of biomarkers of hemoconcentration.

**Patients and Methods:**

We analyzed the early‐ and long‐term effect of SGLT2i empagliflozin on the ePV as assessed by biomarkers of hemoconcentration in a nondiabetic patient with heart failure and reduced ejection fraction (HFrEF) and a nondiabetic patient with heart failure and preserved ejection fraction (HFpEF). The ePV was calculated from hemoglobin and hematocrit levels by Duarte formula and ePV change was calculated by Strauss formula.

**Results:**

The ePV change was −22.56% between baseline and 1 month, and −37.60% between baseline and 12 months follow‐up in a patient with HFrEF, and −6.18% and −16.40% in a patient with HFpEF, respectively.

**Conclusion:**

The early effect of SGLT2i on ePV in patients with heart failure may be monitored by biomarkers of hemoconcentration.

## Graphical abstract

We hypothesized that the early effect of SGLT2i on estimated plasma volume (ePV) may be monitored by biomarkers of hemoconcentration. The ePV change was −22.56% between baseline and 1 month, and −37.60% between baseline and 12 months in patients with HFrEF, and −6.18% and −16.40% in patients with HFpEF, respectively.

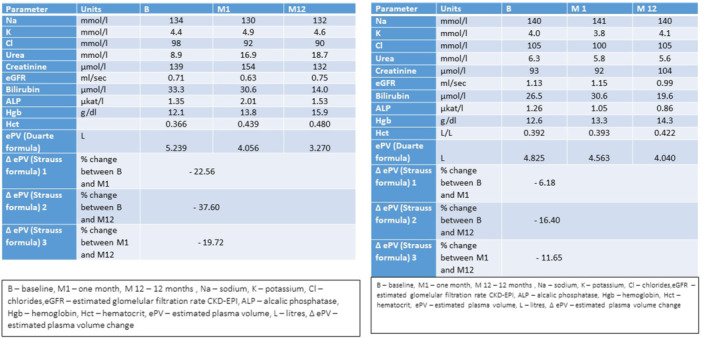

## 1 Introduction

Sodium−glucose co‐transporter‐2 inhibitors (SGLT2i) have been shown to reduce the number of cardiovascular and renal events and the number of heart failure hospitalizations (HFH) in type 2 diabetic patients with a different degree of cardiovascular risk [1, 2, 3]. SGLT2i have shown a beneficial effect on clinical outcome of the patients with established chronic heart failure regardless of the presence of type 2 diabetes and the level of left ventricular ejection fraction [4, 5, 6, 7]. Based on the results of the randomized placebo‐controlled trials, empagliflozin and dapagliflozin are recommended as the fundamental first‐line disease modifying therapy in the patients with heart failure and reduced ejection fraction (HFrEF) in the European and American guidelines for the treatment of heart failure [8, 9]. Empagliflozin and dapagliflozin are recommended as the first‐line therapy in patients with heart failure with preserved ejection fraction (HFpEF) and heart failure with mildly reduced ejection fraction (HFmrEF) in the clinical guidelines update [10].

The reduction of combined cardiovascular mortality and HFH in heart failure patients can be partly explained by the diuretic effect of SGLT2i [11]. Enhanced osmotic diuresis after the introduction of SGLT2i is associated with decrease of estimated plasma volume in type 2 diabetic patients [12, 13].

The change of volume status after SGLT2i leads to the change of hemoglobin and hematocrit. Long‐term change of hemoglobin and hematocrit after SGLT2i is now partly explained by the increase of erythropoetin, which is stimulated by the reduced blood flow in the kidney due to restored tubuloglomerular feedback and by the utilization of ketone bodies [14].

The effect of SGLT2i on estimated plasma volume in nondiabetic patients with heart failure of the different causes of cardiac dysfunction is a subject of the ongoing investigation. Estimation of volume status in elderly patients with heart failure is important. Both hypervolemia and hypovolemia is associated with the risk of adverse outcomes.

We report the early effect of SGLT2i on the estimated plasma volume status assessed by biomarkers of hemoconcentration in a nondiabetic patient with HFrEF and a nondiabetic patient with HFpEF.

## 2 Patients and Methods

A retrospective analysis of the estimated plasma volume status as assessed by biomarkers of hemoconcentration was performed in a patient with advanced HFpEF caused by dilated cardiomyopathy (DCMP) and in a patient with advanced HFpEF caused by hypertrophic cardiomyopathy. Both patients from a tertiary heart failure clinic were followed at least 12 months after the introduction of SGLT2i. Baseline clinic visit with the start of SGLT2i therapy was realized in both cases after discharge after heart failure decompensation. The Strauss formula assessed the change of plasma volume before and after therapy by the change of hemoglobin and hematocrit level at baseline and at follow‐up visits [15].

%plasmavolumechange=100×[hemoglobing/dL(before)/hemoglobing/dL(after)]×[1‐hematocrit(after)/1‐hematocrit(baseline)]−100

Duarte's formula estimated the absolute values of plasma volume using hemoglobin and hematocrit at baseline and at follow‐up visits [16].

ePV=[(1‐hematocrit)]/[hemoglobin(g/dL)]×100

The first patient was a 75‐years‐old nondiabetic male with HFrEF caused by idiopathic DCMP. The diagnosis of DCMP was based on the history of heart failure symptoms that started 7 years ago. The estimated left ventricular ejection fraction as assessed by transthoracic echocardiography was 25%. Coronary arteriography showed normal coronary arteries and cardiac magnetic resonance revealed dilated cardiac chambers with significantly reduced contractility of the left ventricle and absence of the late enhancement as assessed by gadolinium contrast. The patient had a history of long‐term persistent atrial fibrillation with unsuccessful direct current cardioversion. The patient received optimal medical therapy with maximal tolerated doses of beta‐blocker, angiotensin‐converting enzyme inhibitor, mineralocorticoid receptor antagonist and loop diuretic furosemide, and implantable defibrillator (ICD) in primary prevention of sudden death. The patient was referred to a tertiary care heart failure clinic with advanced heart failure symptoms NYHA (New York Heart Association functional classification) class IV after repeated HFH requiring parenteral inotropic and high‐dose diuretic therapy. The patient had severe pulmonary hypertension and was not referred for advanced heart failure therapy because of frailty, comorbidities including chronic kidney disease, and high pulmonary vascular resistance as assessed by a right heart cardiac catheterization study. The hemodynamic study revealed significantly reduced cardiac output (2.5 L/min—litter per minute) and cardiac index (1.29 mL/min/m^2^—litter per minute divided by body surface area). The mean pulmonary artery pressure was 48 mmHg, the mean pulmonary wedge pressure was 30 mmHg, the right atrial pressure was 12 mmHg, and pulmonary vascular resistance was 7 Wood units.

The patient did not tolerate guideline‐directed medical therapy because of low systemic blood pressure and postural symptoms (dizziness and fatigue). His oral pharmacotherapy by that time included a low dose of beta‐blocker (metoprolol succinate 12.5 mg daily), digoxin 0.0625 mg daily, spironolactone 25 mg daily, furosemide 125 mg daily, and dabigatran 110 mg twice daily. The patient did not tolerate any physical activity, he was only able to take one or two steps. He had a nocturnal dyspnea almost every other night. Other symptoms included postural weakness, dizziness, palpitations, bendopnea, and nycturia. The patient had elevated jugular venous pressure (+11 cm), third heart sound, systolic murmur at the apex, basal rales on lung auscultation, hepatomegaly and hepatojugular reflux, and ankle swellings.

After discussion with the patient, the SGLTi empagliflozin was started in 2021 on top of the only tolerated medical therapy. The patient was instructed about hygiene measures. The treatment with empagliflozin 10 mg daily was well tolerated. The treatment was associated with weight reduction within 1 month (−3 kg) and improvement of symptoms. The patient did not report night dyspnea and was able to walk 10 steps at home. The treatment with empagliflozin was associated with the reduced need of HFH. The baseline (before therapy), 1 month, and 12 months after the treatment with SGLT2i laboratory tests were available. The furosemide dose and spironolactone dose has not been changed during 12 months follow‐up.

The second patient was a 77‐years‐old nondiabetic male with HFpEF caused by an advanced stage of nonobstructive hypertrophic cardiomyopathy and permanent atrial fibrillation. The patient was referred to a tertiary care heart failure clinic 6 years ago because of worsening heart failure symptoms. The diagnosis of hypertrophic cardiomyopathy was based on the echocardiography finding of severe septal hypertrophy of the left ventricle (20 mm) without obstruction of the left ventricular output tract. The patient had a normal coronary angiography finding, and cardiac magnetic resonance confirmed septal hypertrophy (22 mm at diastole), left ventricular ejection fraction of 60%, and absence of late gadolinium enhancement. The patient met the criteria for ICD implantation in primary prevention (history of syncope and nonsustained ventricular tachycardia found out at 24 h Holter electrocardiography monitoring. The patient had NYHA class III symptoms and recurrent HFH because of symptoms at rest (NYHA IV). With the evidence of lungs and systemic congestion. The patient was not referred for advanced heart failure therapy because of age. At the last clinic visit before the therapy change, the patient reported dyspnea during regular daily activities (not at rest or at night), low tolerability of exercise, weakness, dizziness, postural vertigo, and leg swellings. After discussion with the patient, the SGLTi empagliflozin 10 mg daily was added to the medical therapy on top of other pharmacotherapy, including maximal tolerated dose of beta‐blocker (bisoprolol 5 mg daily), eplerenone (50 mg daily), and furosemide (187.5 mg daily). The treatment with SGLT2i was well tolerated. The patient's weight decreased by 3 kg within 1 months and exercise tolerability improved. The furosemide dose and the eplerenone dose has not been changed during 12 months follow‐up. The estimated glomerular filtration rate and creatinine level did not change, and hematocrit and hemoglobin levels increased in the first month after gliflozin therapy. The treatment with SGLT2i reduced the risk of HFH in the second patient too. Neither of the two patients received iron replacement therapy or blood transfusion. Neither patient reported any bleeding event during follow‐up.

## 3 Results

The estimated change of plasma volume as assessed by the Strauss formula and the absolute plasma volume values as assessed by the Duarte formula are shown in Tables 1 and 2. The effect of SGLT2 on estimated plasma volume change was more prominent in the first month in a patient with HFrEF than in a patient with HFpEF. The effect of SGLTi on the hemoglobin and hematocrit level persisted 12 months after SGLT2i in both patients.

**Table 1:** Effect of SGLTi on estimated plasma volume in a patient with HFrEF.

Parameter	Units	*B*	M1	M12
Na	mmol/L	134	130	132
K	mmol/L	4.4	4.9	4.6
Cl	mmol/L	98	92	90
Urea	mmol/L	8.9	16.9	18.7
Creatinine	µmol/L	139	154	132
eGFR	mL/s	0.71	0.63	0.75
Bilirubin	µmol/L	33.3	30.6	14.0
ALP	µkat/L	1.35	2.01	1.53
Hgb	g/dL	12.1	13.8	15.9
Hct		0.366	0.439	0.480
ePV (Duarte formula)	L	5.239	4.056	3.270
Δ ePV (Strauss formula) 1	% change between B and M1	−22.56		
Δ ePV (Strauss formula) 2	% change between B and M12	−37.60		
Δ ePV (Strauss formula) 3	% change between M1 and M12	−19.72		

Abbreviations: ALP, alkaline phosphatase; B, baseline; Cl, chlorides; eGFR, estimated glomerular filtration rate CKD‐EPI; ePV, estimated plasma volume; ΔePV, estimated plasma volume change; Hct, hematocrit; Hgb, hemoglobin; K, potassium; L, liters, M1, 1 month; M 12, 12 months; Na, sodium.

**Table 2:** Effect of SGLTi on estimated plasma volume in a patient with HFpEF.

Parameter	Units	*B*	M 1	M 12
Na	mmol/L	140	141	140
K	mmol/L	4.0	3.8	4.1
Cl	mmol/L	105	100	105
Urea	mmol/L	6.3	5.8	5.6
Creatinine	µmol/L	93	92	104
eGFR	mL/s	1.13	1.15	0.99
Bilirubin	µmol/L	26.5	30.6	19.6
ALP	µkat/L	1.26	1.05	0.86
Hgb	g/dL	12.6	13.3	14.3
Hct	L/L	0.392	0.393	0.422
ePV (Duarte formula)	L	4.825	4.563	4.040
Δ ePV (Strauss formula) 1	% change between B and M1	−6.18		
Δ ePV (Strauss formula) 2	% change between B and M12	−16.40		
Δ ePV (Strauss formula) 3	% change between M1 and M12	−11.65		

Abbreviations: ALP, alkaline phosphatase; B, baseline; Cl, chlorides; eGFR, estimated glomerular filtration rate CKD‐EPI; ePV, estimated plasma volume; ΔePV, estimated plasma volume change; Hct, hematocrit; Hgb, hemoglobin; K, potassium; L, liters, M1, 1 month; M 12, 12 months; Na, sodium.

## 4 Discussion

Our results showed the possibility to evaluate the estimated plasma volume change after therapy with SGLT2i in elderly nondiabetic patients with advanced heart failure. The Strauss formula and Duarte formula with hemoglobin concentration and hematocrit level have been used as biomarkers of hemoconcentration [4, 5]. We have shown the decrease of estimated plasma volume after SGLT2i therapy both in an elderly patient with HFrEF caused by DCMP and an elderly patient with HFpEF caused by hypertrophic cardiomyopathy. The enhanced diuresis and reduction of plasma volume leading to decongestion is one of many beneficial effects of SGLT2i explaining the significant effect of the therapy on clinical outcomes in heart failure. The Strauss formula and Duarte formula have been introduced to evaluate the effect of diuretic therapy on decongestion in patients with decompensated heart failure [6]. The treatment with SGLTi is typically associated with a small reduction of glomerular filtration rate and increase of creatinine concentration. The reduction of sodium level is usually not caused by SGLT2i per se, but because of depletion caused by concomitant diuretic therapy. Thus, the diuretic dose should be corrected after initiation of SGLT2i according to volume status, especially in elderly patients.

The estimated plasma volume after the treatment of decompensated heart failure has an important prognostic impact [16]. Biomarkers of hemoconcentration before and after therapy with SGLT2i were used in clinical studies in patients with type 2 diabetes. To our knowledge, the effect of SGLT2i on the estimated plasma volume in elderly nondiabetic patients with heart failure has not been widely investigated. We have also shown a persisting increase of estimated plasma volume at 12 months follow‐up. We are aware about limitations of estimating plasma volume using hemoglobin and hematocrit change. The main limitation of biomarkers of hemoconcentration using hemoglobin and hematocrit is that both may be affected by bleeding events or by blood or iron replacement therapy. The long‐term increase of hematocrit after SGLT2i is explained by the increase in renal erythropoietin [11, 14]. We think the early change of hematocrit and hemoglobin may be used for the estimation of plasma volume after SGLT2i.

## 5 Conclusion

The early effect of SGLT2i on estimated plasma volume change in nondiabetic patients with advanced heart failure may be monitored by the biomarkers of hemoconcentration.

## Ethics Statement

The approval for the study was granted by the Local Board Na Homolce Hospital Ethics Committee on August 4, 2021.

## Consent

Written consent for the study and publication was obtained from the patients.

## Conflicts of Interest

The authors declare no conflicts of interest.

## Data Availability

Data are available on request from the authors.
